# Two-Dimensional CT Tumor Measurement Predicts Pathological Response and Prognosis After Neoadjuvant DCF Therapy in Esophageal Squamous Cell Carcinoma

**DOI:** 10.3390/cancers18121860

**Published:** 2026-06-06

**Authors:** Takahisa Yamaguchi, Koichi Okamoto, Tetsuya Asakawa, Toshikatsu Tsuji, Jun Kinoshita, Shinichi Kadoya, Noriyuki Inaki

**Affiliations:** 1 Department of Gastroenterological Surgery, Ishikawa Prefectural Central Hospital, Kanazawa 920-8530, Ishikawa, Japan; takahisa.1003@gmail.com (T.Y.);; 2 Department of Gastrointestinal Surgery, Kanazawa University, Kanazawa 920-8641, Ishikawa, Japanjunkino0416@gmail.com (J.K.); n.inaki@med.kanazawa-u.ac.jp (N.I.); 3 Department of General and Digestive Surgery, Kanazawa Medical University Hospital, 1-1 Daigaku, Uchinadamachi, Kahoku 920-0293, Ishikawa, Japan

**Keywords:** esophageal squamous cell carcinoma, neoadjuvant chemotherapy, DCF therapy, computed tomography, tumor diameter, pathological response, prognosis

## Abstract

Background/Objective: Evaluating the response to neoadjuvant chemotherapy in esophageal squamous cell carcinoma (ESCC) is challenging because primary tumors are often classified as non-measurable under the RECIST criteria. This study investigated whether two-dimensional computed tomography (CT) measurements predict pathological response and prognosis after neoadjuvant docetaxel, cisplatin, and 5-fluorouracil (DCF) therapy in ESCC. Methods: We retrospectively analyzed 123 patients who underwent radical esophagectomy following DCF therapy at two institutions (April 2011–March 2024). Changes in the short-axis, long-axis, and short-axis × long-axis diameters of the primary tumor after preoperative chemotherapy were measured on contrast-enhanced CT, and the reduction rate for each parameter was assessed. Based on the optimal cutoff values, univariate and multivariate analyses were conducted to identify significant predictors of pathological response. Results: The short-axis × long-axis diameter reduction rate (SLRR) demonstrated the highest area under the curve (0.901), with a cutoff value of 0.55 for predicting pathological response, and it was the only independent predictor in the multivariate analysis (odds ratio, 6.27; *p* = 0.048). In group comparisons using the SLRR cutoff value, patients with a high SLRR had significantly better 3-year recurrence-free survival (67.8% vs. 29.4%, *p* < 0.001) and overall survival (79.5% vs. 46.6%, *p* < 0.001) than those with a low SLRR. Conclusions: The CT-based SLRR independently predicts both pathological response and prognosis after neoadjuvant DCF therapy in ESCC.

## Simple Summary

Assessing the response of esophageal squamous cell carcinoma (ESCC) to neoadjuvant chemotherapy is challenging because primary tumors are often classified as non-measurable under RECIST criteria. This study evaluated whether two-dimensional computed tomography (CT) tumor measurements could predict pathological response and prognosis following neoadjuvant docetaxel, cisplatin, and 5-fluorouracil (DCF) therapy. Among 123 patients who underwent radical esophagectomy after DCF therapy, the reduction rate of the short-axis × long-axis diameter (SLRR) demonstrated the highest discriminatory ability (AUC = 0.901) and was the only independent predictor of pathological response in multivariate analysis (OR = 6.27, *p* = 0.048). Patients with a high SLRR showed significantly better 3-year recurrence-free survival (67.8% vs. 29.4%) and overall survival (79.5% vs. 46.6%) compared to those with a low SLRR. These findings suggest that two-dimensional CT tumor measurement is a clinically practical and powerful tool for predicting treatment response and prognosis in ESCC patients receiving neoadjuvant DCF therapy.

## 1. Introduction

Esophageal cancer is one of the most malignant cancers globally, with more than 0.60 million new cases and 0.54 million deaths per year worldwide [1]. Patients often present with dysphagia and weight loss and are commonly diagnosed by computed tomography (CT), upper gastrointestinal endoscopy, endoscopic ultrasonography and histopathological examination of biopsy specimens. Despite recent advances in surgical techniques including minimally invasive surgery and perioperative management [2,3,4,5], it remains a malignancy with a poor prognosis. Radical esophagectomy is one of the most important treatment modalities for esophageal cancer; however, local control and prevention of recurrence are difficult to achieve with surgery alone, and the usefulness of perioperative adjuvant chemotherapy has been suggested. The efficacy of neoadjuvant chemotherapy (NAC) for esophageal cancer using DCF therapy, which consists of docetaxel, cisplatin, and 5-fluorouracil (5-FU), has been demonstrated in a phase III Japan Clinical Oncology Group (JCOG) trial (JCOG1109) [6]. Based on these results, preoperative DCF therapy has been introduced as the standard treatment for patients with stage II or III esophageal squamous cell carcinoma (ESCC) in Japan. Evaluating the efficacy of chemotherapy prior to surgery is crucial for determining subsequent treatment strategies, and the therapeutic response to chemotherapy is also a significant factor directly associated with patient prognosis [7,8,9].

The Response Evaluation Criteria in Solid Tumors (RECIST) version 1.1 is widely used to assess the efficacy of chemotherapy for solid tumors and is applicable to various tumor types [10]. However, gastrointestinal tumors differ from solid organ tumors in that their size and boundaries are often difficult to evaluate accurately. In particular, the primary lesion of ESCC is frequently regarded as a non-measurable lesion. Furthermore, because some cases of ESCC do not involve lymph node metastasis, the precise degree of tumor shrinkage cannot always be determined by RECIST. To address this issue, the present study evaluated the primary lesion using contrast-enhanced CT (CE-CT) to investigate whether these findings are associated with the therapeutic efficacy of chemotherapy and whether they could serve as prognostic factors.

Recently, several studies have evaluated the therapeutic effect of chemotherapy for ESCC by measuring the primary tumor diameter or tumor volume on CT [11,12,13,14]. However, no studies have evaluated the efficacy of DCF therapy using measurements of the long- and short-axis diameters. In this study, we aimed to clarify whether the long- and short-axis diameters of the primary esophageal tumor measured on CT are associated with pathological response and prognosis in patients with ESCC.

## 2. Materials and Methods

### 2.1. Patients

This study involved 123 patients who underwent radical esophagectomy after DCF therapy for the treatment of ESCC without distant metastases between April 2011 and March 2024 at Ishikawa Prefectural Central Hospital (Kanazawa, Japan) and Kanazawa University Hospital (Kanazawa, Japan). This was a two-institution retrospective cohort study that enrolled patients with resectable clinical stage II/III ESCC treated with NAC, as well as patients with clinical stage IVa locally advanced ESCC treated with induction chemotherapy. Age, sex, TNM stage, and European Cooperative Oncology Group performance status were evaluated by reviewing the patients’ medical records. All patients were staged according to the 11th edition of the Japanese Classification of Esophageal Cancer [15]. This study was approved by the Institutional Review Board of Ishikawa Prefectural Central Hospital (study no: 2533) and the Institutional Review Board of Kanazawa University Hospital (study no: 2019-295).

### 2.2. Chemotherapy Regimen and Surgery

The standard NAC regimen of DCF therapy and the induction chemotherapy regimen are as follows: docetaxel (60–70 mg/m^2^ on day 1), cisplatin (60–70 mg/m^2^ on day 1), and 5-FU (750–800 mg/m^2^ on days 1–5), administered for two or three cycles repeated every 3–4 weeks [16,17]. Subsequent cycles of chemotherapy were discontinued if a serious adverse event was observed. After chemotherapy, all patients were evaluated for resectability by CE-CT, and when the tumor was judged to be resectable, surgery was scheduled. Radical thoracoscopic McKeown esophagectomy with two-field (thoracic and abdominal) or three-field (thoracic, abdominal, and cervical) lymphadenectomy was performed. In cases where a positive surgical margin is suspected, intraoperative frozen section analysis of the margin is routinely performed to confirm R0 resection. Surgery was carried out 4–6 weeks after the last administration of DCF.

### 2.3. Assessment of Primary Tumor Size on CT

**Figure 1:**
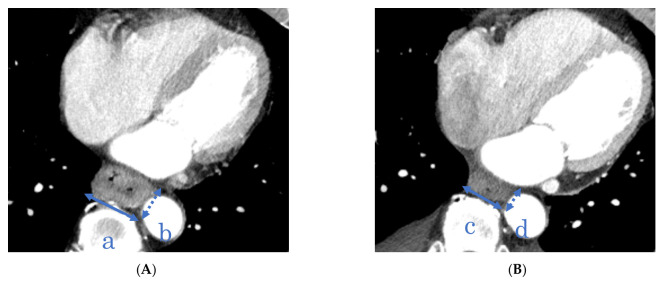
Assessment of long- and short-axis diameters on contrast-enhanced computed tomography before and after chemotherapy. (**A**) Computed tomography image before chemotherapy. The long-axis diameter is indicated by a solid arrow (a), and the short-axis diameter is indicated by a dotted arrow (b). (**B**) Computed tomography image of the same slice after chemotherapy. The long-axis diameter is indicated by a solid arrow (c), and the short-axis diameter is indicated by a dotted arrow (d).

All patients underwent CE-CT before DCF therapy and again 2–3 weeks afterward, with slice thicknesses ranging from 0.5 to 2.5 mm. First, the routine unenhanced scans were performed. Subsequently, Contrast medium was administered intravenously at a dose of 1.5 mg/kg body weight. CT examination was acquired at 30 s as the early contrast phase and at 120 s as the delayed contrast phase. In patients with renal impairment, the dose of contrast medium was adjusted accordingly. Tumor location was determined comprehensively based on differences in enhancement during the early and delayed phases, wall thickness compared with the normal esophageal wall (esophageal wall thickness exceeding 5 mm on axial CT) and the presence of mass formation. Tumor diameter was measured on the horizontal slice showing the thickest part of the tumor among all slices demonstrating the tumor. The long-axis diameter was defined as the maximum distance between the outer margins of the tumor. The short-axis diameter was measured on the same slice as the long-axis diameter and defined as the maximum diameter perpendicular to the long axis. All measurements were performed independently by two investigators (TY and KO), with each investigator conducting one measurement, and the mean value was calculated. Tumor size after chemotherapy was measured at the same CT slice level as before chemotherapy. Details of tumor measurement are shown in Figure 1.

### 2.4. Evaluation of Clinical and Histopathological Response to Chemotherapy

The effect of chemotherapy was evaluated according to RECIST version 1.1 [10]. Clinical response was classified into four categories: complete response (CR), partial response (PR), stable disease (SD), or progressive disease (PD). We defined patients with CR and PR as RECIST responders and those with SD and PD as RECIST non-responders. The histopathological response was categorized into five grades according to the evaluation criteria of the Japanese Classification of Esophageal Cancer, 11th edition [15], as follows: grade 0, no recognizable cytological or histological therapeutic effect; grade 1a, viable cancer cells accounting for two-thirds or more of the tumor tissue; grade 1b, viable cancer cells accounting for one-third or more but less than two-thirds of the tumor tissue; grade 2, viable cancer cells accounting for less than one-third of the tumor tissue; and grade 3, no viable cancer cells. Size reduction in the lesion on CT was assessed using the short-axis diameter, long-axis diameter, and product of the long- and short-axis diameters. The reduction rate for each parameter was calculated as follows: (pretreatment value − post-treatment value)/pretreatment value.

### 2.5. Data Analysis

Univariate and multivariate logistic regression analyses were performed to assess factors predicting the chemotherapeutic effect. Inter-reader agreement between the two investigators was assessed using intraclass correlation coefficient (ICC). Optimal cut-off values for tumor reduction rates on CT measurements were calculated using receiver operating characteristic (ROC) curve analysis based on the maximal Youden index [18]. ROC curves were analyzed in relation to histopathological response to determine their predictive value. We evaluated the predictive accuracy of each parameter by calculating sensitivity. Overall survival (OS) and recurrence-free survival (RFS) were calculated from the initiation of chemotherapy. OS and RFS rates between groups were compared using the log-rank test. Variables showing associations with OS (*p* < 0.1) in the univariate analyses were subsequently included in a multivariate Cox proportional hazards model. A *p*-value of <0.05 was considered statistically significant. All statistical analyses were performed using SPSS Statistics, version 23.0 (IBM Corp., Armonk, NY, USA).

## 3. Results

### 3.1. Patient Characteristics

Table 1 presents the data of 123 patients who underwent esophagectomy after DCF therapy. Their mean age was 64.8 years; 97 patients were male and 26 were female. Clinical stage II, III, and IVa disease was observed in 15 (12.2%), 71 (57.7%), and 37 (30.1%) patients, respectively. DCF chemotherapy was administered for 1 course in 3 patients (2.4%), 2 courses in 74 patients (60.1%), and 3 courses in 46 patients (37.5%). A total of 73 patients (59.3%) experienced grade 3 or higher adverse events according to the Common Terminology Criteria for Adverse Events (CTCAE) version 5.0. According to the RECIST criteria, PR, SD, and PD were observed in 68 (55.3%), 46 (37.4%), and 9 (7.3%) patients, respectively. Histopathological response grades 0, 1a, 1b, 2, and 3 were observed in 19 (15.4%), 44 (35.8%), 15 (12.2%), 30 (24.4%), and 15 (12.2%) patients, respectively. The mean short-axis and long-axis diameters on CT before chemotherapy were 23 mm and 32 mm, respectively. Inter-reader agreement for tumor measurements was evaluated using the ICC. The ICCs for the pre-chemotherapy tumor short-axis and long-axis diameters were 0.782 and 0.841, respectively. The ICCs for the post-chemotherapy tumor short-axis and long-axis diameters were 0.715 and 0.741, respectively.

### 3.2. Optimal Cutoff Values of Reduction Rates of CT Parameters Using ROC Curves and Area Under the Curve (AUC)

**Figure 2:**
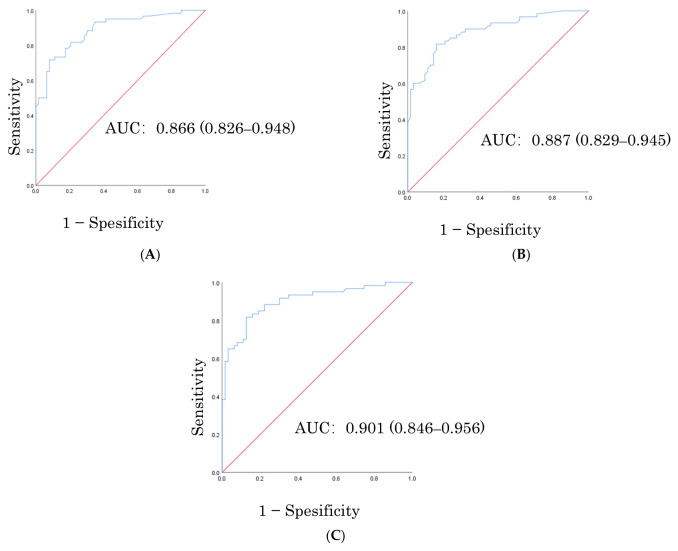
Receiver operating characteristic curves for pathological response and (**A**) short-axis reduction rate, (**B**) long-axis reduction rate, (**C**) and short-axis × long-axis reduction rate. AUC, area under the receiver operating characteristic curve.

We constructed ROC curves and calculated the AUC to determine the optimal cutoff values for the short-axis reduction rate (SRR), long-axis reduction rate (LRR), and short-axis × long-axis reduction rate (SLRR) in predicting the efficacy of chemotherapy (Figure 2A–C). The AUC for the SRR was 0.866 (95% confidence interval [CI], 0.826–0.948), with a cutoff value of 0.33. The AUC for the LRR was 0.887 (95% CI, 0.829–0.945), with a cutoff value of 0.40. The AUC for the SLRR was 0.901 (95% CI, 0.846–0.956), with a cutoff value of 0.55. These values were used for subsequent analyses. An SRR of ≤0.33 was defined as a low SRR, and an SRR of >0.33 was defined as a high SRR. Similarly, an LRR of ≤0.40 was defined as a low LRR, and an LRR of >0.40 was defined as a high LRR. An SLRR of ≤0.55 was defined as a low SLRR, and an SLRR of >0.55 was defined as a high SLRR.

### 3.3. Relationship Between Clinicopathological Characteristics and Pathological Response to Chemotherapy

In the univariate analysis, we examined the clinicopathological factors associated with the pathological response to chemotherapy (Table 2). For subsequent analyses, pathological response grades 0–1a were classified as the low pathological response group and grades 1b–3 were classified as the high pathological response group. Pathological response was not associated with age, sex, body mass index, performance status, tumor location, or clinical stage. Although more patients with a high pathological response were observed among RECIST responders, the difference was not statistically significant. By contrast, significantly more patients with a high pathological response were observed among those with a high SRR (78.3% vs. 22.2%, *p* < 0.001), high LRR (81.6% vs. 20.6%, *p* < 0.001), and high SLRR (88.3% vs. 23.8%, *p* < 0.001). The sensitivity of chemotherapy response based on RECIST was 57.7%, whereas the sensitivity using the SLRR was 82.1%.

### 3.4. Multivariate Analysis for Predicting the Pathological Response to Chemotherapy

Table 3 shows the results of logistic regression analysis for predicting the pathological response to chemotherapy. Multivariate analysis showed that the RECIST response (odds ratio [OR], 1.55; 95% CI, 0.210–1.980; *p* = 0.444), SRR (OR, 2.130; 95% CI, 0.540–8.440; *p* = 0.280), and LRR (OR, 2.900; 95% CI, 0.551–15.30; *p* = 0.209) were not significant factors. By contrast, the SLRR was the only parameter that showed a statistically significant association as a predictor of the therapeutic effect (OR, 6.270; 95% CI, 1.215–40.40; *p* = 0.048).

### 3.5. Long-Term Outcomes

**Figure 3:**
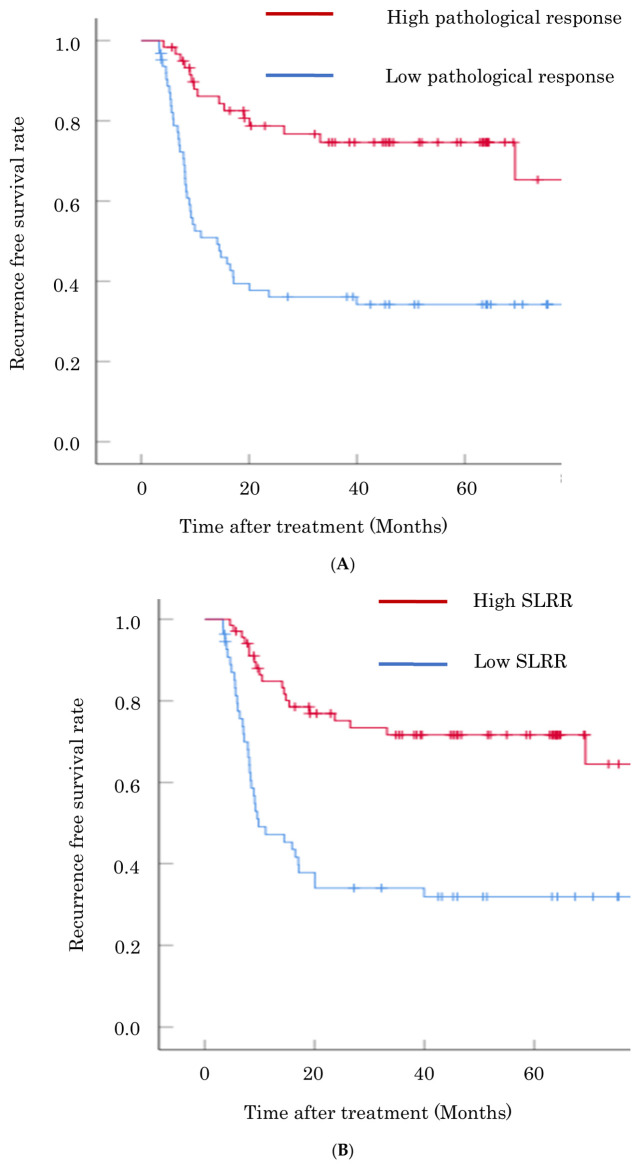
Kaplan–Meier curves of recurrence-free survival according to (**A**) pathological response (high vs. low) and (**B**) short-axis × long-axis reduction rate (SLRR > 0.55 vs. SLRR ≤ 0.55).

**Figure 4:**
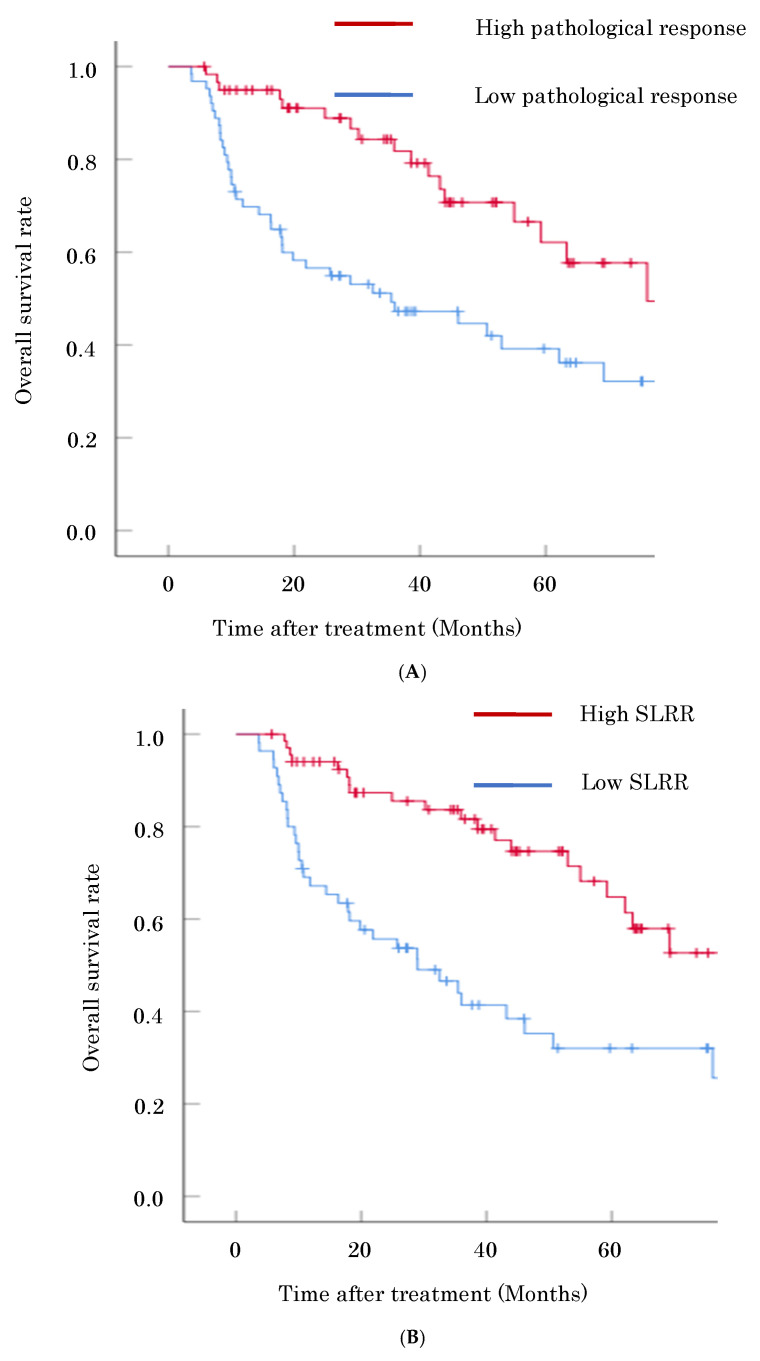
Kaplan–Meier curves of overall survival according to (**A**) pathological response (high vs. low) and (**B**) short-axis × long-axis reduction rate (SLRR > 0.55 vs. SLRR ≤ 0.55).

The median follow-up period was 36.3 months. There were 48 death events and 54 relapse events during the follow-up period. Survival curves for RFS according to pathological response and SLRR are shown in Figure 3A,B. The 3-year RFS rate in the high pathological response group was significantly better than that in the low pathological response group (69.5% vs. 33.8%, *p* < 0.001). In addition, the 3-year RFS in the high-SLRR group was significantly better than that in the low-SLRR group (67.8% vs. 29.4%, *p* < 0.001). Figure 4 shows that OS was similar to RFS, with better OS observed in the high pathological response group and high-SLRR group than in the low pathological response group (70.7% vs. 51.2%, *p* < 0.001) and low-SLRR group (79.5% vs. 46.6%, *p* < 0.001), respectively.

The results of the univariate and multivariate analyses of OS are shown in Table 4. In the univariate analysis, body mass index, clinical stage, RECIST response, and high SLRR were significant prognostic factors. In the multivariate analysis, clinical stage (hazard ratio [HR], 2.247; 95% CI, 0.253–0.783; *p* = 0.005), RECIST responder (HR, 1.814; 95% CI, 1.004–3.277; *p* = 0.049), and high SLRR (HR, 2.526; 95% CI, 1.355–4.708; *p* = 0.004) were identified as independent prognostic factors, with high SLRR showing the highest HR.

## 4. Discussion

This study investigated whether changes in the long- and short-axis diameters of the primary esophageal tumor measured on CE-CT are associated with pathological response and prognosis following neoadjuvant DCF therapy in patients with ESCC. Our data showed that the reduction rate of the short-axis × long-axis diameter is an independent CT-based predictor of pathological response and an independent prognostic factor for both RFS and OS.

RECIST is the most widely used framework for evaluating the efficacy of chemotherapy in solid tumors. However, its applicability to the primary lesion of ESCC is inherently problematic. Because the primary esophageal tumor, present within a tubular organ, is often considered an “unmeasurable” lesion under the RECIST criteria, the exact degree of tumor shrinkage cannot always be quantified. This limitation is particularly significant in the current clinical landscape, where DCF therapy has become widely adopted as the standard neoadjuvant regimen for stage II/III ESCC based on the results of the JCOG1109 trial [6]. In the present study, the predictive accuracy of chemotherapy response based on RECIST was 57.7%, whereas CT-based evaluation demonstrated a higher predictive rate of 82.1%.

To date, various modalities have been reported for evaluating the efficacy of chemotherapy in ESCC. The usefulness of 18F-fluorodeoxyglucose positron emission tomography (FDG-PET) has also been reported as a modality for assessing the therapeutic effect of chemotherapy in ESCC [19,20,21,22]. A decrease in FDG uptake during neoadjuvant treatment correlates with chemotherapeutic effect and prognosis; however, FDG-PET is not a convenient examination to be performed repeatedly before and after chemotherapy in routine clinical practice. In addition, methods that evaluate treatment response by measuring tumor volume on CT have been reported [12,13,14], but this approach requires imaging analysis software for volumetric assessment and is therefore not widely applicable in daily practice. The CT examinations evaluated in our study are indispensable for assessing tumor shrinkage and determining surgical indications before and after chemotherapy. Moreover, because assessment can be performed simply by measuring tumor diameter, this method is considered both convenient and clinically useful.

In this study, among the three CT parameters evaluated (SRR, LRR, and SLRR), SLRR showed the highest AUC (0.901; 95% CI, 0.846–0.956) and was the only independent predictor of pathological response in the multivariate analysis (OR, 6.270; 95% CI, 1.215–40.40; *p* = 0.048). This finding is consistent with recent reports suggesting that two-dimensional tumor measurements provide more informative data than assessments based on a single unidirectional tumor diameter [23,24]. In our previous study, we demonstrated that DCF therapy, compared with 5-FU + cisplatin therapy, significantly improved the degree of stenosis at the primary esophageal cancer site, along with a reduction in the dysphagia score, which reflects clinical symptoms. The evaluation employed not only the stenotic length and width of the narrowest segment at the primary site on fluoroscopy, but also two-dimensional changes in luminal area, suggesting a correlation between improvement in stenosis and pathological response [25]. Sato et al. [7] demonstrated that assessment based on the 12th edition of the Japanese Classification of Esophageal Cancer, which incorporates endoscopic evaluation of the primary site, allows for more precise prognostic stratification of chemotherapy response than the RECIST criteria in patients who underwent neoadjuvant DCF therapy. This classification defines measurable lesions as primary esophageal cancer with a long-axis diameter exceeding 20 mm on CT slices and evaluates the macroscopic response at the primary site. These reports support the findings of our study.

Taniyama et al. [11] conducted a multicenter study of patients with ESCC receiving NAC and demonstrated that the short-axis diameter of the primary tumor was independently correlated with pathological response in multivariate analysis and significantly associated with both OS and disease-free survival. Matsumoto et al. [26] similarly reported that the reduction rate of the short-axis diameter of the primary esophageal tumor was a better predictor of pathological response than the long-axis diameter or tumor area, with an AUC of 0.781 and an optimal cutoff of 22%. The present study extends these findings by demonstrating that combining both axes into a single area-based metric further improves predictive performance. Using the product of the short- and long-axis diameters allows a more comprehensive two-dimensional assessment of tumor regression. This may explain why the SRR alone (AUC 0.873) provided nearly the same amount of information as the LRR alone (AUC 0.887), and why the combined parameter (SLRR) demonstrated the highest discriminatory ability.

Pathological response to NAC is well established as a major determinant of long-term prognosis in ESCC. In the present cohort, the 3-year RFS was 33.8% in the low pathological response group and 69.5% in the high pathological response group. These outcomes are consistent with multiple studies demonstrating that patients achieving a favorable histopathological response after NAC have substantially better RFS and OS than those with minimal response [8,27,28]. Notably, the prognostic significance of the area reduction rate on CT (SLRR) was almost equivalent to that of pathological response. Patients with a high SLRR showed a 3-year RFS of 67.8% compared with 29.4% in those with low SLRR, and this parameter was confirmed as an independent prognostic factor with the highest HR among significant variables. This finding suggests that preoperative CT measurements can serve as a reliable surrogate for pathological tumor regression before histopathological assessment is available. The ability to predict prognosis non-invasively and prior to surgery has important clinical implications for risk stratification, patient counseling, and the early identification of candidates for intensified adjuvant treatment.

Although the study population comprised patients with resectable esophageal cancer, the same evaluation method could potentially be applied to assess the efficacy of chemotherapy in unresectable cases, contributing to the prediction of pathological response. In Japan, where NAC is the standard treatment, the CROC trial was conducted to evaluate the effectiveness of a chemotherapy selection strategy based on patients’ response to DCF therapy, in which either definitive chemoradiotherapy or surgery was chosen accordingly [29]. After induction DCF therapy, a marked clinical response was observed in 58.4% of patients, and 89.8% of these responders received definitive chemoradiotherapy and achieved clinical CR. The primary endpoint, the 1-year progression-free survival rate, was 89.8%, and the 3-year OS rate was 83.7%. Patients showing a limited partial response or poor response were scheduled for curative surgery, and the 3-year organ preservation rate was 45.3%. These findings provide important evidence supporting the advancement of future organ-preserving strategies and further highlight the potential utility of this study approach for predicting treatment response on CT imaging.

The therapeutic response to chemotherapy in esophageal cancer varies markedly among patients, and the identification of reliable biomarkers capable of predicting treatment efficacy remains a major unmet clinical need. A wide range of potential biomarkers has been investigated, including epidermal growth factor receptor expression [30], *ERCC1* mRNA expression [31], tumor-infiltrating lymphocytes [32], and inflammation-based indicators. In addition, several easily measurable and non-invasive parameters, such as the neutrophil-to-lymphocyte ratio, have been reported as predictive markers of chemotherapy efficacy, and, together with CT-based evaluations, are expected to attract increasing attention in the future. However, none of these have yet been established as standard biomarkers, and their clinical utility as standalone predictive factors remains limited; further investigation is therefore warranted.

This study has some limitations. First, this was a retrospective analysis conducted at two institutions, and selection bias inherent to this design cannot be completely excluded. Second, the study population included patients treated with induction chemotherapy for cStage IVa disease. This may introduce heterogeneity in treatment outcomes, potentially resulting in bias; however, the inclusion of locally advanced cases treated with induction chemotherapy also increases the real-world applicability of this study. Third, CT measurement of esophageal tumors is inherently subject to interobserver variability. In accordance with previous reports [11,12,14], regions with wall thickening of ≥5 mm on axial CT images were considered tumor lesions, and tumor diameter was measured accordingly. Although this measurement approach is straightforward and easy to implement, it may be susceptible to inter-reader variability. ICCs for pre-chemotherapy tumor diameters demonstrated good reliability, and those for post-chemotherapy tumor diameters demonstrated moderate reliability. Based on these findings, the reproducibility was generally within an acceptable range; however, standardizing imaging slice selection and the definition of tumor boundaries is important for improving accuracy. Fourth, pathological response of grade 1b or higher was defined as an effective response to chemotherapy in this study. However, some studies have adopted grade 2 or higher as the threshold for defining an effective response, which may lead to different results depending on the criteria used. We considered it clinically appropriate to include grade 1b as part of an effective response, given that grade 1b has been shown to correlate with prognosis [33], and this classification reflects actual clinical practice. Fifth, the relatively small number of patients and the limited number of pathological complete responders (grade 3: 12.2%) may limit statistical power for subgroup analyses. Multicenter prospective validation in larger cohorts is warranted.

## 5. Conclusions

The reduction rate of the short-axis × long-axis diameter product, as measured on CE-CT before and after DCF therapy, is an independent CT-based predictor of pathological response to neoadjuvant DCF therapy in ESCC and an independent prognostic factor for both RFS and OS. These findings support the routine incorporation of two-dimensional primary tumor measurement into pre- and post-chemotherapy CT evaluation protocols for ESCC. This approach provides clinically actionable information that standard RECIST criteria fail to capture and can assist surgeons and oncologists in risk stratification, treatment modification decisions, and perioperative prognostication—all without requiring additional imaging resources beyond standard CE-CT.

## Figures and Tables

**Table 1:** Clinicopathological data.

Variables		Value
Age (years)	Mean ± std	64.8 ± 7.72
Sex	Male	97 (78.9)
	Female	26 (21.1)
Performance status	0	111 (90.2)
	1	12 (9.8)
Tumor location	Ce	4 (3.3)
	Ut	23 (18.7)
	Mt	53 (43.1)
	Lt	37 (30.1)
	Ae	6 (4.8)
Short-axis diameter on CT before chemotherapy (mm)	Average ± std	23 ± 7.88
Long-axis diameter on CT before chemotherapy (mm)	Average ± std	32 ± 11.0
cStage (11th JES)	II	15 (12.2)
	III	71 (57.7)
	IVa	37 (30.1)
Number of chemotherapy cycles	1	3 (2.4)
	2	74 (60.1)
	3	46 (37.5)
Adverse events (CTCAE ver5.0) ≥3	YES	73 (59.3)
	No	40 (40.7)
RECIST	PR	68 (55.3)
	SD	46 (37.4)
	PD	9 (7.3)
Histopathological response	0	19 (15.4)
	1a	44 (35.8)
	1b	15 (12.2)
	2	30 (24.4)
	3	15 (12.2)

CTCAE, Common Terminology Criteria for Adverse Events; std, standard deviation; PR, partial response; SD, stable disease; PD, progressive disease.

**Table 2:** Clinicopathological characteristics according to pathological response of the primary tumor to chemotherapy.

Variables		High Pathological Response (*n* = 60)	Low Pathological Response (*n* = 63)	*p*-Value
Age	Median	67	65	0.499
	(range)	(46–79)	(47–77)	
Sex	Male	47 (78.3)	50 (79.4)	0.889
	Female	13 (21.7)	13 (20.6)	
BMI	>21.0 kg/m^2^	34 (56.7)	30 (47.6)	0.315
	≤21.0 kg/m^2^	26 (43.3)	33 (52.4)	
Performance status	0	53 (88.3)	58 (92.1)	0.486
	1	7 (11.7)	5 (7.9)	
Tumor location	Ce	1 (1.7)	2 (3.2)	0.755
	Ut	12 (20.0)	12 (19.0)	
	Mt	28 (46.7)	25 (39.7)	
	Lt/Ae	19 (31.6)	24 (38.1)	
cStage (11th JES)	II	8 (13.3)	7 (11.1)	0.772
	III	35 (58.4)	36 (57.2)	
	IVa	17 (28.3)	20 (31.7)	
RECIST	PR	38 (63.3)	30 (47.6)	0.080
	SD + PD	22 (36.7)	33 (52.4)	
SRR	>0.33	47 (78.3)	14 (22.2)	* <0.001
	≤0.33	13 (21.7)	49 (77.8)	
LRR	>0.4	49 (81.6)	13 (20.6)	* <0.001
	≤0.4	11 (18.4)	50 (79.4)	
SLRR	>0.55	53 (88.3)	15 (23.8)	* <0.001
	≤0.55	7 (11.7)	48 (66.2)	

SRR, short-axis reduction rate (pre-chemotherapy tumor short-axis diameter − post-chemotherapy tumor short-axis diameter) divided by the pre-chemotherapy tumor short-axis diameter; LRR, long-axis reduction rate (pre-chemotherapy tumor long-axis diameter − post-chemotherapy tumor long-axis diameter) divided by the pre-chemotherapy tumor long-axis diameter; SLRR, short-axis × long-axis reduction rate (pre-chemotherapy short-axis × long-axis diameter − post-chemotherapy short-axis × long-axis diameter) divided by the pre-chemotherapy short-axis × long-axis diameter. * *p* < 0.05.

**Table 3:** Multivariate analysis for predicting pathological response to chemotherapy.

Variables		OR	95% CI	*p*-Value
RECIST	PR SD + PD	1.550	0.210–1.980	0.444
SRR	>0.33 ≤0.33	2.130	0.540–8.440	0.280
LRR	>0.4 ≤0.4	2.900	0.551–15.30	0.209
SLRR	>0.55 ≤0.55	6.270	1.215–40.40	* 0.048

SRR, short-axis reduction rate (pre-chemotherapy tumor short-axis diameter − post-chemotherapy tumor short-axis diameter) divided by the pre-chemotherapy tumor short-axis diameter; LRR, long-axis reduction rate (pre-chemotherapy tumor long-axis diameter − post-chemotherapy tumor long-axis diameter) divided by the pre-chemotherapy tumor long-axis diameter; SLRR, short-axis × long-axis reduction rate (pre-chemotherapy short-axis × long-axis diameter − post-chemotherapy short-axis × long-axis diameter) divided by the pre-chemotherapy short-axis × long-axis diameter. * *p* < 0.05.

**Table 4:** Cox regression analysis of overall survival.

Variables	Univariate Analysis		Multivariate Analysis	
	HR (95% CI)	*p*-Value	HR (95% CI)	*p*-Value
Age		0.515		
<70	1			
≥70	1.21 (0.463–1.471)			
Sex		0.568		
Male	1			
Female	0.827 (0.426–1.606)			
BMI		0.025		0.065
>21.0 kg/m^2^	1		1	
≤21.0 kg/m^2^	0.468 (0.266–0.824)		0.556 (0.314–1.116)	
Performance status		0.402		
0	1			
1	1.380 (0.650–2.927)			
Tumor location		0.152		
Ce/Ut	1			
Mt/Lt/Ae	0.868 (0.852–2.809)			
cStage (11th JES)		* 0.016		* 0.005
II/III	1		1	
IVa	1.942 (1.133–3.327)		2.247 (1.277–3.952)	
RECIST		* 0.018		* 0.049
PR	1		1	
SD/PD	1.908 (1.119–3.257)		1.814 (1.004–3.277)	
SLRR		* <0.001		* 0.004
>0.55	1		1	
≤0.55	2.915 (1.675–5.05)		2.526 (1.355–4.708)	

HR, hazard ratio; CI, confidence interval; BMI, body mass index; PR, partial response; SD, stable disease; PD, progressive disease; SLRR, short-axis × long-axis reduction rate. * *p* < 0.05.

## Data Availability

The datasets used during the current study available from the corresponding author on reasonable request.
